# Common Lung Microbiome Identified among Mechanically Ventilated Surgical Patients

**DOI:** 10.1371/journal.pone.0166313

**Published:** 2016-11-29

**Authors:** Ashley D. Smith, Yan Zhang, Robert C. Barber, Christian T. Minshall, Ryan M. Huebinger, Michael S. Allen

**Affiliations:** 1 Department of Cell Biology and Immunology, University of North Texas Health Science Center, Fort Worth, Texas, United States of America; 2 Center for Biosafety and Biosecurity, University of North Texas Health Science Center, Fort Worth, Texas, United States of America; 3 Department of Molecular and Medical Genetics, University of North Texas Health Science Center, Fort Worth, Texas, United States of America; 4 Department of Surgery, University of Texas Southwestern Medical Center, Dallas, Texas, United States of America; AOU Città della Salute e della Scienza di Torino, ITALY

## Abstract

The examination of the pulmonary microbiome in patients with non-chronic disease states has not been extensively examined. Traditional culture based screening methods are often unable to identify bacteria from bronchoalveolar lavage samples. The advancement of next-generation sequencing technologies allows for a culture-independent molecular based analysis to determine the microbial composition in the lung of this patient population. For this study, the Ion Torrent PGM system was used to assess the microbial complexity of culture negative bronchoalveolar lavage samples. A group of samples were identified that all displayed high diversity and similar relative abundance of bacteria. This group consisted of *Hydrogenophaga*, unclassified Bacteroidetes, *Pedobacter*, *Thauera*, and *Acinetobacter*. These bacteria may be representative of a common non-pathogenic pulmonary microbiome associated within this population of patients.

## Introduction

While numerous studies have examined the gut microbiome in relation to disease, the lung microbiome by comparison has not been extensively examined. Culture-independent molecular methods enabled by so-called next-generation or massively parallel sequencing (MPS) technologies have allowed for the advancement of studies of the lung and its resident microbial populations. Bacteria found in the lungs can be classified into two categories: potentially pathogenic microorganisms (PMMs) and non-potentially pathogenic microorganisms (non-PMMs) which are sometimes referred to as “normal respiratory tract flora” and are not typically associated with infections in non-immunocompromised individuals. Well-known pulmonary PMMs include *Pseudomonas aeruginosa*, *Haemophilus* spp., *Staphylococcus aureus*, *Streptococcus pneumonia*, *Moraxella catarrhalis*, and members of the Enterobacteriaceae. A few examples of non-PMMs include: *Streptococcus* viridans group, *Candida* spp., *Corynebacterium* spp., and *Neisseria* spp. [1].

Previous pulmonary microbiome research centered largely on chronic disease states such as cystic fibrosis, chronic obstructive pulmonary disorder (COPD), asthma, and smoking (reviewed in [2]). These studies have demonstrated that the lung microbiome varies between and within different stages of disease. For example, different microorganisms and levels of diversity are found in a patient whose asthma or COPD is stable versus those undergoing an acute exacerbation of the disease. This also holds true in patients with cystic fibrosis and HIV [3, 4]. For example, an increase in Proteobacteria within the pulmonary microbiome was found in HIV and asthma patients as compared to their respective controls [5, 6]. Several recent studies have examined the microbiome in patients that are intubated [7, 8]. Kelly et al. noted that microbial diversity was initially lower than healthy controls shortly after intubation. Additionally, microbial diversity decreased over the length of intubation [8].

Initial studies of the lung microbiome concluded that the lung had no distinct bacterial community and that bacteria found there could most likely be attributed to contamination from the upper respiratory tract [9]. Subsequent investigations have shown that while microaspiration is normal in healthy individuals, bacteria do reside within the lungs [10, 11]. However, studies have also shown that there is overlap between the bacteria residing in the upper and lower respiratory tracts [11, 12]. *Prevotella* and *Veillonella* are two of the most common genera that reside in the lungs and nasopharynx [13]. Other common genera found within the lung microbiome include *Haemophilus* and *Streptococcus* [11].

Prior to MPS, the microbial content of the lungs, particularly during infection, were identified using standard culture-based methods. This is still the primary method of pathogen identification for pneumonia diagnosis in the clinical pathology lab. However, bacterial cultivation in a lab setting is sometimes difficult [14, 15]. This is especially true for pathogens such as *Mycobacterium* spp. or *Mycoplasma* spp. that display slow rates of growth or grow poorly on traditional differential media [16, 17]. One study reported that an etiologic agent could not be identified in approximately 46% of cases of community-acquired pneumonia [18], highlighting the inadequacy of current culture-based techniques in the diagnosis of pneumonia. Patients undergoing mechanical ventilation are particularly at increased risk for developing pneumonia [19]. Consequently, as part of the standard of care for ventilated patients, some hospitals screen bronchoalveolar lavage (BAL) samples for infectious microbes. In this study, we utilize MPS to examine culture negative BAL samples from mechanically ventilated surgical patients. We report that a number of patients in this study with diverse underlying medical conditions were found to share a common lung microbial community composition. The results shed light on the composition of the lung microbiome in this patient cohort and highlight the potential for molecular based diagnostics for determining lung infections.

## Materials and Methods

### Clinical Samples

Clinical bronchoalveolar lavage (BAL) samples were collected from mechanically ventilated patients in the surgical ICU at Parkland Memorial Hospital. Patient information from BAL samples used in this study are presented in Table 1. Specific clinical data of each BAL including antibiotic usage and ventilator events are presented in Table 2. BALs were collected using an unprotected BAL catheter in accordance with standard operating procedures developed by the large-scale collaborative project, “Inflammation and the Host Response to Injury” [20]. As part of the standard of care, BALs are performed on patients that remain ventilated for over 36 hours (screening) or those with a Clinical Pulmonary Infection Score (CPIS) greater than or equal to 6 [21]. As part of a de-escalation antibiotic management clinical protocol, administration of antibiotics is stopped if the BAL culture results are negative. Subsequently, based upon this protocol culture negative BAL patients are clinically classified as patients with Systemic inflammatory response syndrome (SIRS) and not pneumonia. This protocol was approved by the Institutional Review Boards at University of Texas Southwestern Medical Center (UTSW) and University of North Texas Health Science Center. Written consent for the BAL procedure was obtained by the practicing physician and documented in the patient’s medical record. Research samples utilized in this study were collected under waived consent for discarded material from standard of care BAL procedure. Collection and utilization of the samples for research utilization were approved by the UTSW IRB.

**Table 1:** Demographic Information for Bronchoalveolar Lavage Samples.

BAL ID #	Age	Gender	Race	Days Post Injury of BAL	Injury	Expired
32	49	M	White	6	Fall from Ladder	No
34	25	M	White	6	MVC (Motor Vehicle Collision)	No
42	20	M	Asian	2	GSW (Gunshot wound) to head	Yes- Gunshot wound
69	30	M	Hispanic	2	MPC (Motor Person Collision)	No
70	29	M	White	14	GSW	Yes- Bradycardia after long hospital stay from Gunshot wound
133	61	F	Black	3	Bowel resection	Yes- Intra-abdominal abscess s/p multiple bowel resections, multiple washouts, complicated by fistula formations, malnutrition, poor wound healing
137	53	F	Black	3	Mandibular abcess and tooth extraction	No
164	30	M	Hispanic	2	Motor Cycle Crash (MCC), Traumatic Brain Injury (TBI)	Yes- Traumatic brain injury
186	26	M	Hispanic	2	GSW abdomen	No
189	18	F	White	2	MVC	No
196	54	M	Hispanic	12	MVC	No
197	61	M	Black	6	Colon Cancer, perforated Colon	No
201	50	M	Black	2	Day Surgery (Back), subsequent abdominal pain and surgery	No
208	20	F	Black	2	MVC with severe TBI	No
209	40	M	Asian	4	Early stage rectal cancer, surgery to remove tumor	Yes- Sepsis, respiratory failure

**Table 2:** Prior antibiotic administration and clinical information of BAL samples.

BAL ID #	Reason for BAL	On ABX at time of BAL	Antibiotic administration(dose frequency)	Clinical presentation
32	Diagnostic	Yes	6 doses of 1500mg Vancomycin (every 12 hours); 9 doses 3.375g piperacillin/tazobactam (every 8 hours) prior to BAL	CXR bilateral chest infiltrate; acute respiratory failure
34	Diagnostic	No	3 doses of 200mg ciprofloxacin (daily) prior to BAL	Previous BAL 4 days prior grew 70k CFU *Enterobacter cloacae*; CXR Lung opacity clearing since previous BAL
42	Screening	No	One dose 600mg clindamycin 48 hour prior to BAL	No VE at time of Bal
69	Diagnostic	No	No antibiotics prior to BAL	No VE at time of BAL; subsequent BAL 3 days later had 10K CFU *Staphylococcus aureus*, some infiltrate on CXR at time of second BAL
70	Diagnostic	Yes	7 doses of 1750mg vancomycin (every 12 hours), last dose 5 days prior to BAL; 21 doses of 3.37g piperacillin/tazobactam (every 8 hours) for 8 days preceding BAL	ABX for positive *Enterobacter* urine culture; CPIS 8
133	Screening	Yes	3 doses of 600mg ciprofloxacin (daily) prior to BAL	ABX for abdominal abscess; CXR minimal lower lung infiltrates; extubated 2 days after BAL
137	Screening	Yes	7 doses of 3.375g piperacillin/tazobactam (every 8hours) 2 days prior to BAL; 2 doses 1000mg vancomycin (every 12 hours) prior to BAL	admitted to hospital 3 days prior to BAL with possible Pneumonia(cough, sputum production, tachypnea)
164	Screening	Yes	3 doses of 600mg clindamycin (every 12 hours) immediately prior to BAL	No associated Ventilator events
186	Screening	No	No antibiotics prior to BAL	Intubated for >2 weeks with no ventilator events
189	Screening	Yes	2 doses of 400mg gentamicin (daily) prior to BAL	CXR clear at time of BAL
196	Diagnostic	No	No antibiotics prior to BAL	No associated Ventilator events; extubated 2 days after BAL
197	Screening	Yes	24 doses of 3.375g piperacillin/tazobactam (every 8 hours) completed 13 days before BAL; 39 doses 500mg metronidazole (3x per day) immediately prior to BAL; 7 doses of 1250mg vancomycin (every 12 hr) immediately prior to BAL; one doses 100mg micafungin 24hr prior to BAL	No associated Ventilator events; extubated 2 days after BAL
201	Screening	Yes	6 doses piperacillin/tazobactam (every 8 hours) prior to BAL; one dose 500mg azithroymycin 48 hours prior to BAL; one dose 1500mg Vancomycin 24 hours prior to BAL	No associated Ventilator events
208	Screening	No	One dose 120mg gentamycin 120mg 5 days prior to BAL; 7 doses of 80mg gentamycin (daily) last dose 2 days before BAL	No associated Ventilator events
209	Screening	Yes	2 doses of 400mg flucanozole (every 24 hrs) immediately before BAL; one dose 80mg gentamycin 3 days before BAL; 7 doses of 3.375g piperacillin/tazobactam (every 8 hours) immediately before BAL	No significant symptoms at time of BAL; subsequent CXR showed increasing infiltrate and edema; Positive *Pseudomonas* infection in subsequent BALs(4 days-40kCFU and 9 days-100kCFU after initial BAL)

### BAL Sample Processing

Upon collection, a portion of the raw BAL fluid was placed in a polypropylene tube and placed at 4°C. The remaining BAL fluid was submitted to the Parkland Memorial Hospital Clinical Pathology Laboratory for microbiological identification as part of the standard of care. The raw BAL samples retained for research were picked up twice daily from the Surgical ICU and transported to the UTSW Surgery Core BSL2+ laboratory where each sample was given a study unique identification number that was used as part of the de-identification process. A one-ml aliquot of raw BAL fluid was placed in a cryovial and stored at -80°C for microbial community analysis.

### Pathology Laboratory Protocol

The Parkland Memorial Hospital Clinical Pathology Laboratory performed a semi-quantitative culture by using a 1μl disposable loop to plate raw BAL fluid onto Chocolate agar, MacConkey agar and Trypticase Soy Agar with 5% sheep blood. A Gram stain was also prepared by smearing raw BAL fluid onto a glass slide. The BAL samples were pelleted by centrifugation at 3,000 x *g* for 15 minutes and the sediment was processed for acid-fast bacillus culture. A sterile transfer pipet was then used to inoculate the following fungal culture media: Sabouraud Dextrose Agar Inhibitory Mold Agar and Brain Heart Infusion with blood.

### DNA Isolation

DNA was extracted using the MasterPure DNA purification kit (Epicentre, Chicago, IL). The cell pellet was processed according to the manufacturer’s protocol except as noted below. BAL samples were removed from -80°C storage and allowed to thaw on ice. Once thawed, sputum was removed and the samples were pelleted by centrifugation at 15,000 x *g* for 15 minutes. Briefly, a solution of Proteinase K (50μg/μl) diluted in Tissue and Cell Lysis Solution was added to each cell pellet and vortexed thoroughly to suspend the cells. The samples were then incubated at 65°C for 15 min. Next, samples were allowed to cool before addition of RNase A (5μg/μl) and a second incubation at 37°C for 30 min. After incubation, DNA was precipitated then resuspended in C6 Tris-based buffer from the PowerSoil DNA Isolation kit (Mo Bio). Extracted DNA was maintained frozen at -20°C until ready to use.

### 16S rRNA gene PCR Amplification and Processing

Extracted DNA was amplified using AccuPrime™ Taq DNA Polymerase High Fidelity (Life Technologies, Grand Island, NY). The V4 hypervariable region of 16S rRNA gene was targeted using 515 forward (GTGCCAGCMGCCGCGGTAA) and 806 reverse (GGACTACHVGGGTWTCTAAT) [22]. Sample specific barcodes and Ion Torrent adapters were synthesized with the forward or reverse primers following the IonXpress barcode design (Life Technologies, Carlsbad, CA). The 25μl total volume reaction mix consisted of: 2.5μl 10x AccuPrime™ PCR Buffer II, 1μl each of 10μM Ion Torrent specific barcoded Forward and Reverse Primer, 1μl 50mM MgSO_4_, 0.1μl AccuPrime™ Taq Polymerase High Fidelity, with molecular grade water and template DNA making up the remaining 17.8μl. The reaction was amplified on a Bio-Rad C1000 thermocycler under the following conditions: 94°C for 2 min, followed by 30 cycles of 94°C for 15s, 52° C for 15s and 68°C for 20s before a final extension at 68°C for 5 min. The resulting ~350 bp fragments were visualized on a 1.5% agarose gel with Ethidium Bromide. PCR reactions were performed in triplicate for each sample.

Triplicate barcoded 16S rRNA PCR products were pooled for purification with Agencourt AMpure XP Reagent (Beckman Coulter Genomics, Danvers, MA) according to the Ion Torrent protocol. Purified PCR products were assessed for DNA size, molarity, and quality using an Agilent DNA 7500 or High Sensitivity kit and read on an Agilent Bioanalyzer 2100 (Agilent Technologies, Santa Clara, CA). Molarity measurements obtained were used to dilute the samples to equimolar concentrations.

### Ion Torrent Sequencing

Purified amplicons were pooled and diluted so that the final concentration of the DNA library was 26 pM in low TE. The sample was then subjected to the three step Ion Torrent One Touch System according to the manufacturer’s protocol. Briefly, the sample was amplified by emulsion PCR using the Ion Torrent One Touch II System and 400 bp chemistry. The resultant ion sphere particles (ISPs) were enriched on the Ion Torrent ES. The enriched ISPs were then loaded onto a 316 v2 sequencing chip for semi-conductor sequencing on an Ion Torrent Personal Genome Machine.

### Quantification of 16S rRNA gene copy number

Droplet Digital PCR (ddPCR) on a QX200 ddPCR system (Bio-Rad, Hercules, CA) was used to quantify the number of 16S rRNA gene copies present within a given BAL sample. Prior to droplet generation, a room temperature reaction mix was made consisting of: 11μl of 2x QX200 ddPCR EvaGreen Supermix, 0.44μl 515F primer (10μM), 0.44μl 806R primer (10 μM), and 9.02 μl H_2_O with 1.1μl of template DNA for a total of 22μl per sample. 20μl of the reaction mix and QX200 Droplet Generation Oil for EvaGreen (70μl) were transferred to the appropriate wells in a DG8 cartridge. After droplet generation, ~40μl of formed droplets were transferred into a 96-well PCR plate that was then heat sealed with a foil seal. The samples were amplified by PCR in a Bio-Rad C1000 thermocycler under the following conditions: 95°C for 5 min, followed by 40 cycles of 95°C for 30s and 52°C for 2 min before final signal stabilization at 4°C for 5 min and 90°C for another 5 min. Droplets were read in the QX200 droplet reader and final copies/20μl sample measurements were calculated by the QuantaSoft software (BioRad).

### Data Analysis

Data analysis was performed using a mothur data-analysis pipeline. Briefly, DNA sequences belonging to individual BAL samples were identified by sample-specific barcodes. Primers and barcodes were trimmed, short and (<100 bp) low quality sequences (qaverage = 20) were also removed from the dataset. The aligned sequences were further denoised using a precluster algorithm and screened for chimeras using UCHIME [23]. Sequences were grouped into operational taxonomic units (OTUs) with a cutoff of 97% similarity. Shannon diversity and Chao1 richness were calculated at the 97% similarity level. The Ribosomal Database Project (RDP) was used to perform a taxonomic classification of sequences with a minimum 80% confidence [24]. The microbial communities among the BAL samples were compared using UniFrac analysis [25]. Principal Coordinate Analysis (PCoA) and hierarchical cluster analysis (Unweighted Pair Group Method with Arithmetic Mean algorithm) based on both unweighted and weighted UniFrac distances were also conducted to display the relationship among the BAL samples. The UniFrac, PCoA and hierarchical cluster analysis were performed with the mothur pipeline [26].

Data Availability: The sequences in this study are deposited in the NCBI sequence read archive (SRA) database under the accession number SRP082546 (Bioproject PRJNA339755).

## Results

### Relative abundance of culture negative BAL

**Fig 1:**
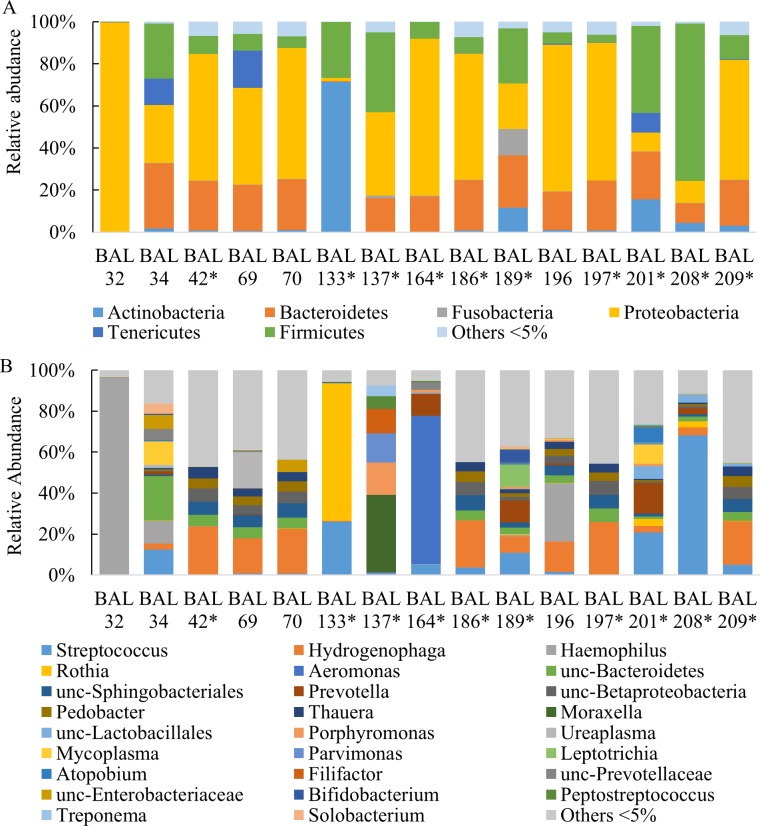
Relative Abundance of Culture Negative BAL Samples. Raw BAL from culture negative BAL samples were sequenced on the Ion Torrent PGM by amplifying the v4 region of the 16S rRNA gene. Analysis of the sequences were completed with the mothur pipeline using the RDP reference database. The number of sequences of bacteria were converted to percentages of the total. Only taxon comprising >5% of a sample were graphed; anything <5% is listed as other. Phylum (A) and genus (B) level taxonomy are presented. Unc-denotes that the sequence was unclassified at the genus level, and the lowest level identified is listed instead. *Screening BALs.

Ion Torrent PGM sequencing was used to evaluate the microbial community within the lungs of mechanically ventilated surgical patients. Fifteen unique patient samples previously determined to be culture negative were sequenced. Of the fifteen samples, fourteen did not grow bacteria by standard plating techniques at Parkland Hospital and results of the other sample was reported as normal “respiratory tract flora” (BAL133) (Table 1). A total of 1,227,232 sequences were used for taxonomic classification with a range of 29,880 to 143,487 sequences per sample (Table 3). Sequences were classified to the lowest taxonomic designation possible; most at the genus level. The relative abundance of bacteria by taxonomic classification level is detailed in Fig 1. Culture negative BAL samples were dominated by three phyla: Proteobacteria (46.98%), Firmicutes (19.14%), and Bacteroidetes (18.51%), In addition, Actinobacteria comprised a majority of BAL133 and smaller proportions of BALs 189, and 201(Fig 1A). Differences between individual samples were more apparent at genus level classification where *Streptococcus*, *Hydrogenophaga*, and *Haemophilus* were among the most common genera present in samples (Fig 1B). Most noteworthy was the observation that seven BAL samples (BALs 42, 69, 70, 186, 196, 197, and 209) were comprised of highly similar mixtures of bacterial genera including *Hydrogenaphaga*, unclassified Sphingobacteriales, unclassified Betaproteobacteria, unclassified Bacteroidetes, *Pedobacter*, *Thauera*, *Haemophilus*, and *Actinobacter* among others. These samples differed from the other eight samples in that they appeared to be less dominated by one or more potential pathogenic microorganisms (PPMs). Additionally, several of the BAL samples (BALs 69, 186 and 196) of this group received no antibiotics prior to collection of BAL fluid (Table 2).

**Table 3:** Total Number of Observed Bacterial Sequences from Culture Negative BAL.

BAL ID #	Number of sequence reads
32	52,154
34	29,880
42*	82,728
69	74,117
70	64,639
133*	99,040
137*	58,499
164*	103,107
186*	89,426
189*	73,684
196	98,240
197*	77,000
201*	92,188
208*	143,487
209*	87,493
**All BAL Samples**	**1,227,232**

*screening BAL.

### Assessing the microbiome diversity of culture negative BAL

**Fig 2:**
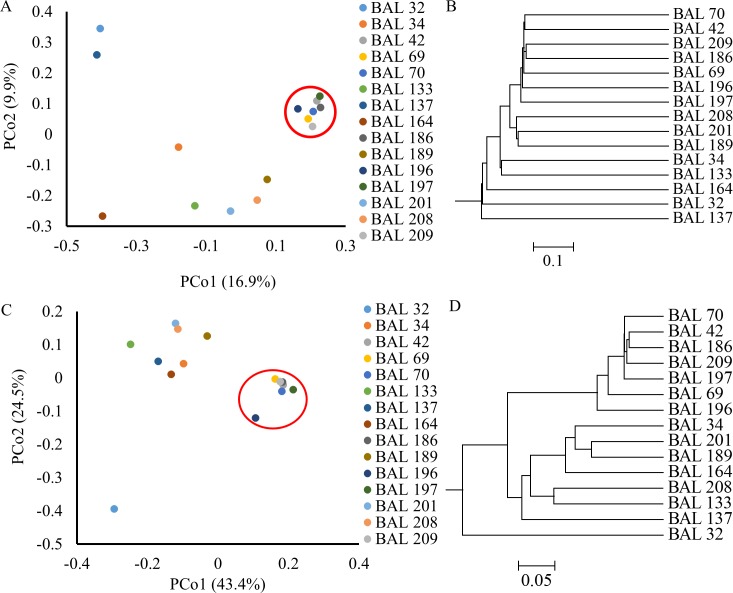
Distance Analyses of Culture Negative BAL Samples. UniFrac analysis was used to analyze the phylogenetic distances between in communities in each BAL sample, this was visualized by unweighted (Fig 2A-B) and weighted (Fig 2C-D) Principles Coordinates Analysis (PCoA, Fig 2A and C) and a dendrogram trees (Fig 2B-C). Each PCoA axis (PCo1 or PCo2) describes the percentage of variation between samples that the axis is able to explain. Common samples that cluster together are circled in red.

In order to compare the microbial community in the BAL samples, a UniFrac distance metric was applied and visualized by principal coordinate analysis (PCoA) and hierarchical analysis (Fig 2). BALs 42, 69, 70, 186, 196, 197, and 209 all displayed highly similar genera (Fig 1B) and clustered together in both unweighted and weighted analyses (Fig 2).

**Fig 3:**
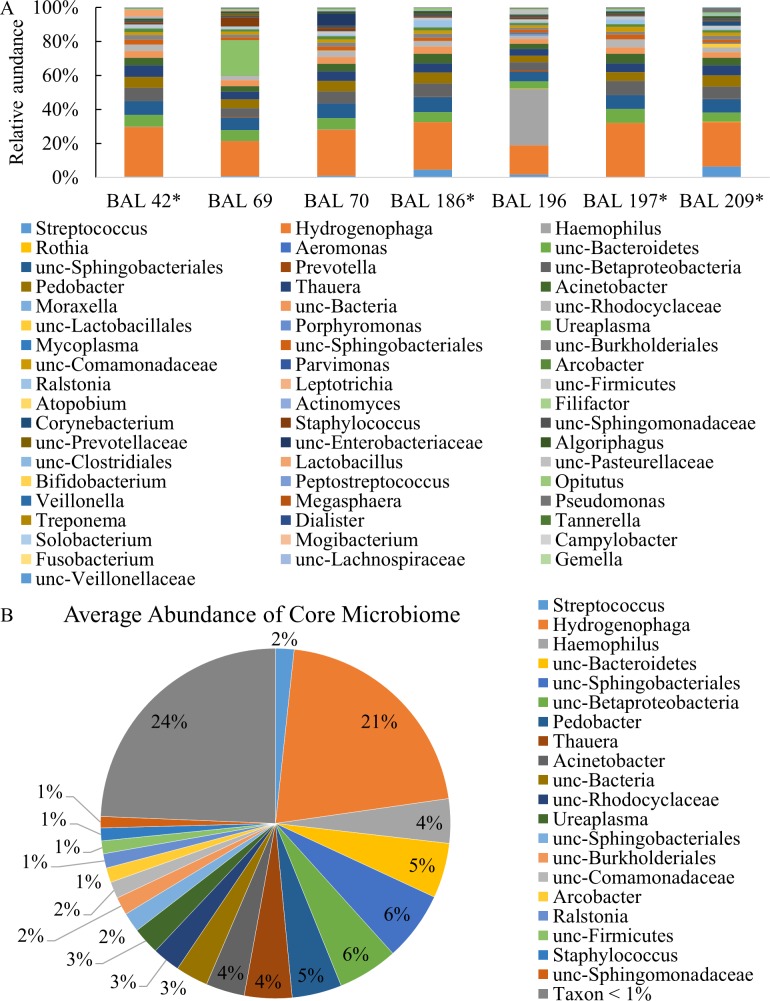
Examination of Relative Abundance of Culture negative BAL Samples with common profile. Genus level relative abundance of all common BAL samples (A), and the average of all common samples together (B). Genera depicted represent ≥1%. * Screening BALs

The genus-level relative abundance data of the screening BALs were selected and plotted together to illustrate the similarities in bacterial composition (Fig 3A). An average composite of the bacterial communities is shown in Fig 3B. Of the 55 total genera identified in the common microbiome samples, 20 were present at average levels ≥1%. *Hydrogenophaga* comprised the largest percentage (21%). Most other genera were present in the range of 1–6%, including *Haemophilus*, *Streptococcus*, *Staphylococcus*, *Acinetobacter*, and others that were not able to be classified down to the genus level. The final 24% of the common microbiome group included 35 other genera sequenced at an average abundance of less than 1% in the samples.

**Fig 4:**
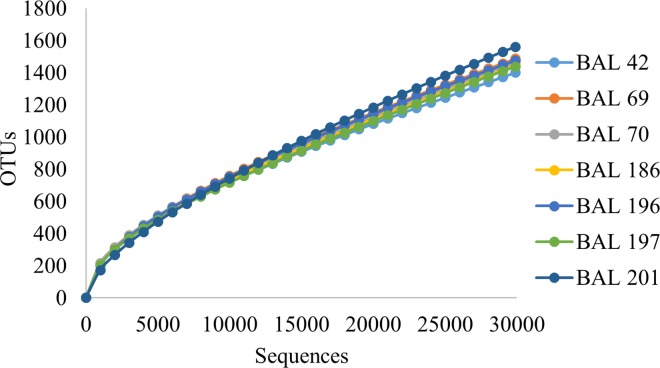
Alpha Diversity Estimates. Rarefaction curve showing number of sequences generated versus the number of operational taxonomic units (OTUs) identified in each BAL sample at a cutoff of 97% sequence similarity.

Alpha diversity measurements were also used to investigate the specific characteristics of the common microbiome BAL samples. The rarefaction curve (Fig 4) indicated sufficient sampling depth for each BAL sample and showed high similarity among common group members. Shannon and Chao1indices were used to assess the diversity and richness of the BAL samples (Table 4). The common group exhibited higher levels of diversity for mean number of OTUs (p = 0.007), Shannon diversity (p = 0.006), and Chao1 richness (p = 0.0001). However, when stratified by antibiotic utilization at time of BAL diversity metrics were not statistically different (observed number of OTUs (p = 0.45), Shannon diversity (p = 0.313), and Chao1 richness (p = 0.746)).

**Table 4:** Quantification of 16S gene copies in Culture Negative BAL.

BAL ID #	Pathology Lab Results	16S rRNA gene copies/ μl ± SEM
32	No Growth	297,733±14,133
34	No Growth	187,667±5,333
42	No Growth	7,700 ± 503
69	No Growth	19,373 ± 397
70	No Growth	141,067 ± 1,827
133	Respiratory Tract Flora	undetermined
137	No Growth	835,800±29,631
164	No Growth	1,345,333±21,333
186	No Growth	24,267 ± 624
189	No Growth	21,840±706
196	No Growth	10,860 ± 1,244
197	No Growth	6,160 ± 136
201	No Growth	252,533±9,354
208	No Growth	93,333±6,967
209	No Growth	134,000 ± 5,108

### Quantification of 16S rRNA gene copies in BAL samples

The amount of bacteria present may have a significant effect on patient symptoms, treatment and outcome. Droplet digital PCR was therefore used to quantify the number of 16S rRNA gene copies in the microbiome BAL samples. The number of 16S rRNA gene copies in each culture negative BAL sample ranged from 6,160 (BAL 197) to 1,345,333 copies/μl (BAL 164) (Table 4). The mean 16S rRNA gene copy number was higher in the non-common BAL samples (433,462copies/μl) versus the common profile (49,061 copies/μl), although it was not statistically significant (p = 0.079). (Table 5) Additionally, when stratified on antibiotic usage at time of BAL, the number of 16S rRNA gene copies were not statistically different (p = 0.096).

**Table 5:** Alpha Diversity Estimates of Culture Negative BAL Samples

BAL ID #	Number of observed OTUs^a^	Shannon	Chao1
32	783	0.64	2526
34	923	3.21	2871
42	1400	4.30	6,638
69	1486	4.15	6,167
70	1461	4.35	6,936
133	916	1.31	3,062
137	876	2.33	4,332
164	639	1.49	3,047
186	1447	4.25	6,815
189	1451	4.39	5,573
196	1475	3.88	5,201
197	1438	4.12	6,474
201	1560	3.91	5,200
208	1059	2.10	3,268
209	1464	4.42	6,603

^a^ at 97% sequence similarity level

## Discussion

Next-generation sequencing of culture negative BAL from mechanically ventilated surgical patients revealed the presence of a distinct group of bacterial genera that represented a common microbiome profile among this patient cohort. This common group exhibited higher diversity and contained many of the same microorganisms in similar proportions as indicated by UniFrac distance analysis. This group also had a reduced bacterial abundance as measured by 16S rRNA gene copies in each sample. Although some of the patients in this cohort were exhibiting signs of pneumonia at time of BAL, many of the samples in the common group were screening BALs (i.e. patients were not necessarily experiencing signs and symptoms of pneumonia at the time the BAL was performed) (Table 2). During the time of this study, screening BALs were performed as part of the standard of care at Parkland Memorial Hospital. Any patient who was on mechanical ventilation for over 72 hours received one every few days to preemptively screen for possible infection. Currently there are few published studies on the pulmonary microbiome in healthy individuals, and while the finding of *Streptococcus spp*. is consistent with other groups’ findings, a majority of results reported here are unique [8, 9, 27, 28]. For example, some of the genera identified in large proportions by others are observed here at much lower levels. Additionally, *Hydrogenophaga*, *Pedobacter* and *Thauera* were identified, which have not been previously reported [28]. Differences in bacterial identification can be attributed to many factors including differences in DNA extraction techniques, sequencing platforms, primer/target selection, or potentially related to geographic location of patients (i.e. region of the U.S.). Further analysis will be required to determine the commonality of these bacterial groups among patients in other regions.

Quantification of bacteria is a particularly important factor in diagnostics and can be used as an early diagnostic indicator of infection. Knowing the standard baseline number of 16S rRNA gene copies for the average microbiome may allow clinicians to quickly identify an increase in bacterial load. The mean copy number of approximately 65,000 copies/μl measured here could indicate how many bacteria may be present in the core pulmonary microbiome. BALs 70 and 209 did, however, exhibit approximately ten times more 16S copies per microliter compared to the other 5 samples. This could be attributed to early infection which has not yet led to an apparent dysbiosis; however, given the high similarity among the community profiles, it is more likely that differences in number represent variation in BAL sampling success.

This group of patients is characterized by a diverse array of antibiotic utilization prior to BAL and also ventilator associated events at the time of BAL (Table 2). Several of the patients received no antibiotics prior to collection of the BAL (BAL 69, 186, and 196). These BAL samples were part of the common microbiome profile cluster identified in this study. Of these three, one patient (BAL69) had a subsequent BAL three days later which had 10,000 CFU of *Staphylococcus aureus* growth and some chest opacity (based upon chest X-ray) at the time of follow-up BAL. The other patients that did not receive antibiotics prior to BAL sampling showed no signs of pneumonia over their intubation. Patient BAL186 was intubated for over 2 weeks without a pneumonia ventilator event and patient BAL197 was extubated two days after initial BAL sampling. Other patient’s samples that exhibited the common microbiome profile received varying doses of antibiotics prior to BAL. The group of patients with the common lung microbiome profile cluster had higher levels of diversity relative to samples that were not a part of this cluster.

It should also be noted that techniques based on 16S rRNA gene copy number are only semi-quantitative. Different bacterial species possess varying numbers of 16S rRNA gene copies per genome (e.g. *E*. *coli* contains seven copies in its genome, while *Streptococcus pneumoniae* contains four, and *Mycoplasma pneumoniae* has only one copy) [29, 30]. However, with knowledge of the relative abundance and total 16S rRNA gene copy number, it is theoretically possible to calculate the true number of pathogens present within a sample, provided variability in sampling efficiency can be addressed. One possible means to address this would be to quantify the pathogen DNA using a target-specific gene marker, and normalize those data to results derived from a secondary control targeting a frequently occurring commensal microbe. However, much more research would be required to determine what commensal microbe(s) would serve as a suitable benchmark and how the abundance of these differ across patients and over time. Additionally, PCR-based techniques are generally limited in their ability to discriminate live bacterial cells from dead DNA fragments [31], although recent advances may allow for selective PCR amplification of viable cells[32].

Finally, the patient population presented in this work is one that is not implicitly healthy. However, since the need for ventilation in this cohort was attributed primarily to traumatic injuries, it is reasonable to assume that the common microbiome identified here may be similar to that of a healthy individual. Given the sample size and diverse array of samples in the current study, we were unable to draw a conclusion regarding effects of antibiotics on the microbiome profile of the lung. The identification of a non-pathogenic pulmonary microbiome profile may assist in the clinical diagnosis of pneumonia and allow clinicians to distinguish patients with a microbiome that would lead to the development of pneumonia versus those patients that do not require immediate treatment.
